# The niche of dermal graft to reconstruct a complex pressure injury wound in sacral region: A case report

**DOI:** 10.1097/MD.0000000000036617

**Published:** 2023-12-22

**Authors:** Te-Wei Cheng, Yun-Nan Lin, Su-Shin Lee, Yur-Ren Kuo

**Affiliations:** a Department of General Medicine, Taipei Medical University Hospital, Taipei, Taiwan; b Division of Plastic Surgery, Department of Surgery, Kaohsiung Medical University Hospital, Kaohsiung Medical University, Kaohsiung, Taiwan; c School of Post-Baccalaureate Medicine, College of Medicine, Kaohsiung Medical University, Kaohsiung, Taiwan.

**Keywords:** dermal grafting, pressure-induced injury, pressure ulcer

## Abstract

**Rationale:**

Pressure ulcers are a common health issue, particularly among elderly and bedridden patients who are vulnerable to pressure injuries in the sacral region. Currently, free flap and local flap surgeries are the gold standard procedures for the reconstruction of such injuries. However, the recurrence rate of flap surgery appears to be high. In this context, we presented a case involving a sacral pressure ulcer reconstructed with dermal grafting.

**Patient concerns:**

A 59-year-old male with a medical history of hepatitis C, brain hemorrhage, hydrocephalus, and multiple fractures presented with a sacral ulcer. Owing to the patient’s history of recurrent pressure injuries and the challenges associated with postoperative wound care, the patient and his family were hesitant to proceed with flap surgery.

**Diagnoses:**

The patient was diagnosed with a stage IV pressure ulcer measuring 4 cm × 4 cm in size in the sacral region, according to the National Pressure Ulcer Advisory Panel staging system.

**Interventions:**

Before surgery, the patient received standard wound care with dressing for 4 months, along with short-term oral antibiotics due to a positive wound culture for *Pseudomonas aeruginosa*. During the surgery, a dermal graft with a size of 35 cm^2^ and a thickness of 0.014 inches was harvested from the patient’s left thigh. The graft was then secured to the wound bed.

**Outcomes:**

Although the dermal graft failed with sloughing after 1 week, the wound bed showed improvement with granulation. After 1.5 months, the wound area had decreased to half of its original size, and the wound eventually healed after 3.5 months.

**Lessons:**

Dermal grafts have a niche in reconstructing pressure injury wounds in the sacral region, because of the relative ease of wound care and additional benefits even in cases where the graft fails.

## 1. Introduction

Pressure ulcers, also known as pressure sores, are a global health concern. This pressure-induced injury is a consequence of prolonged external force and ischemia.^[1]^ Besides the elderly, populations who are bedridden or dependent on wheelchairs usually suffer from bedsores due to immobilization, impaired sensation, or malnutrition.^[2]^ According to international pressure ulcer prevalence surveys conducted between 1989 and 2005, the overall prevalence of pressure ulcers was approximately 15% in healthcare facilities. Moreover, the sacrum was the most common region, accounting for about 30% of cases.^[3]^

For patients with stage III and IV injuries, the reconstructive ladder includes primary wound closure, skin grafting, regional flaps, and free flaps.^[4]^ Currently, flaps are the gold standard for sacral pressure sores.^[5]^ On the other hand, for lesions on the lower limbs, skin grafts such as split-thickness skin grafting are recommended. These clinical approaches were supported by Schryvers et al^[6]^ whose 20-year retrospective study found that flaps could tolerate pressure for pressure injury wound on pelvic region including sacral, ischial, and trochanteric sites. However, according to Keys et al, the recurrence rate of pressure ulcers after flap surgery is up to 39%, with an average follow-up period of 4.4 years.^[7]^

Therefore, in this case, we presented an innovative method of dermal grafting to reconstruct a complex and ulcerative wound in the sacral region in a patient with pressure injury.

## 2. Case report

This was a 59-year-old male with underlying hepatitis C without medication and a history of intracranial hemorrhage, subdural hemorrhage, hydrocephalus treated with ventriculoperitoneal shunt and lumbar peritoneal shunt, left 5th and 6th rib fractures, right distal radius fracture treated with open reduction and internal fixation and short arm splint, and L1 compression fracture under vertebroplasty. Substance use in alcoholism and amphetamine abuse was noted. He had been bedridden for years and presented to our plastic surgery outpatient department due to ulcers in the sacral region for 4 months.

**Figure 1.**
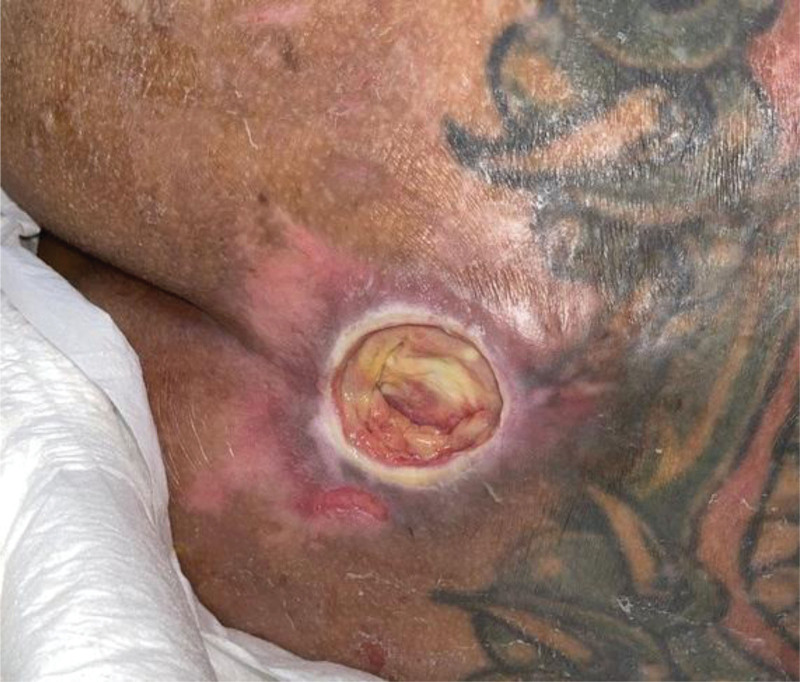
The pressure ulcer at the first visit was stage IV, as there was full-thickness skin and tissue loss with exposed fascia. It measured 4 cm × 4 cm in size and was located in the sacral region.

According to the National Pressure Ulcer Advisory Panel system, the patient was diagnosed with stage IV pressure ulcers in the sacral region, measuring 4 cm × 4 cm in size (Fig. 1). Standard wound care was provided using Biodyne ointment (China Chemical & Pharmaceutical Co., Ltd., Taiwan), Purilon gel (Coloplast Co., Ltd., UK), and AQUACEL Foam (ConvaTec Inc., NC). The patient underwent routine outpatient department follow-up and was prescribed oral antibiotics because of a positive wound culture for *Pseudomonas aeruginosa*. In addition, bedside debridement was performed following the recommendations of the plastic surgeons. The preoperative wound was a stage III pressure ulcer with granulation tissue in the sacral region.

The study was conducted in accordance with the Declaration of Helsinki. Informed consent statement was obtained from the patient for publication of this study. Our study is a case report, and data sharing is not applicable to this article since no datasets were generated or analyzed during the current study.

## 3. Operation technique

**Figure 2.**
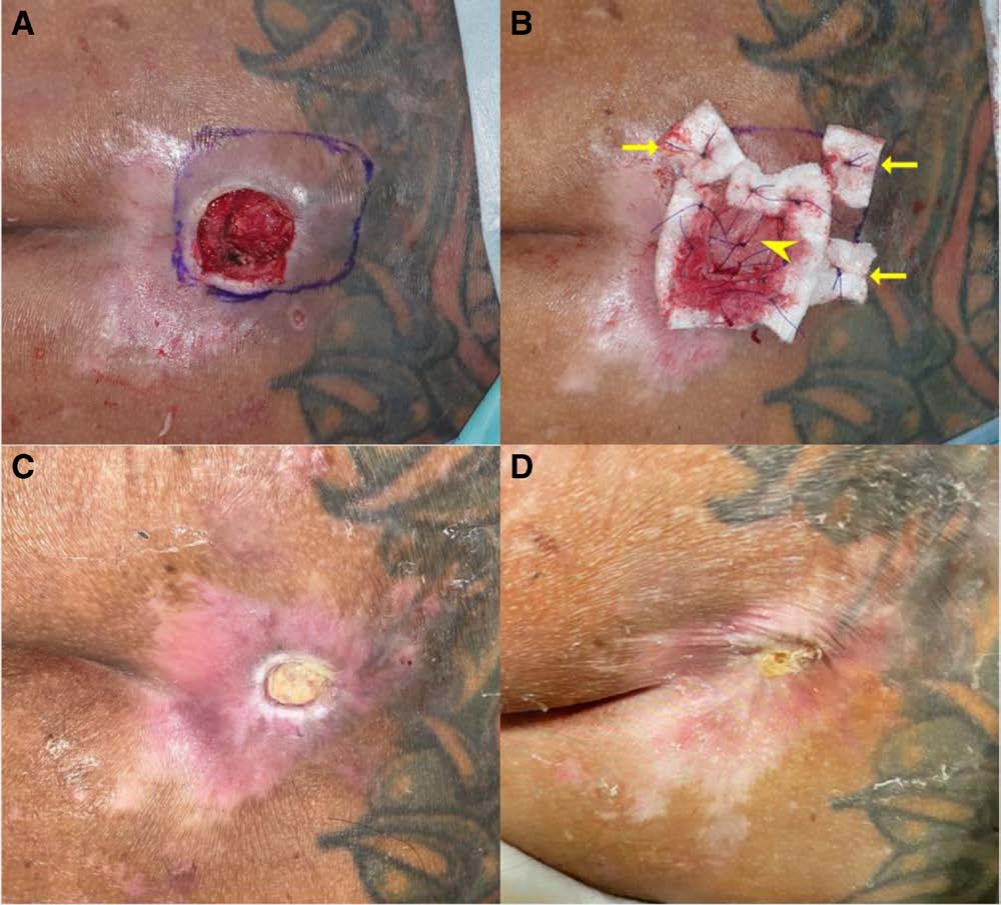
(A) The recipient site of the pressure ulcers in the sacral region, stage III, with granulation tissue on the day of the operation, after debridement. (B) The recipient site on the day of the operation after dermal grafting with anchoring sutures and pledgets with SI-AID (➤ dermal graft; ➞ SI-AID). (C) A picture taken at the 2-month follow-up revealed that the sloughing graft improved the wound bed. (D) A picture taken at the 3.5-month follow-up showed that the wound had healed.

**Figure 3.**
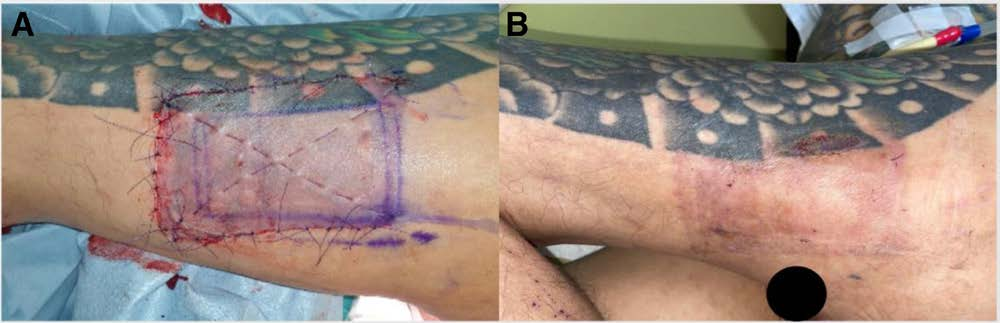
(A) The dermal graft was harvested after the epidermal flap was elevated, with a thickness of 0.012 inches. A dermal graft with a size of 35 cm^2^ and thickness of 0.014 inches was harvested from the left thigh. (B) A picture taken at the 2-month follow-up showed that the donor site was healing well.

An epidermal flap with a thickness of 0.012 inches was elevated using a Zimmer dermatome. A dermal graft measuring 35 cm^2^ and 0.014 inches in size was harvested from the left thigh and transplanted into the marked area (Fig. 2A). Anchoring sutures were performed using pledgets with SI-AID (ALCARE CO., Ltd, Japan) (Fig. 2B). The epidermal flap was then reattached to the underlying tissue at the donor site (Fig. 3A). After the operation, wound care was provided using Biodyne ointment and AQUACEL Foam. The patient was discharged from the hospital after receiving prophylactic antibiotics for 3 days. He continued to receive follow-up care at the plastic surgery outpatient department on a weekly basis. The donor site healed well without any complications (Fig. 3B). Although the graft failed with slough after 1 week, the wound bed improved with granulation tissue, and the wound area decreased by half in size after 2 months (Fig. 2C). The wound healed completely after 3.5 months (Fig. 2D).

## 4. Discussion

The pathophysiology of pressure ulcers is related to sustained external force on body prominence.^[2]^ It has been reported that a pressure of approximately 32 mm Hg over the arterial capillary or around 8 to 12 mm Hg over the venous capillary would cause occlusion of blood flow, which results in hypoxia in local tissue.^[8]^ Those who were bedridden, chair-bound, had impaired nutrition, or had a history of cerebrovascular accident were vulnerable to such pressure-induced injury.^[9]^ Conservative treatment is suitable for low grade pressure ulcers (stages I to II), while reconstructive surgery is indicated to the patient with wound deep or hard to heal (stages III to IV).^[5]^ The recurrence rate seemed to be higher in patients undergoing skin grafting, which accounted for 75% of the total recurrence, compared to that of patients receiving flaps, if not avoiding weight bearing or no neurologic or functional recovery.^[10]^ However, even with the flap procedure, the recurrence rate was around 16.8% to 42% (Table 1). Bamba et al reported that the complication rate after flap reconstruction was 58.7% in a study of patients who underwent surgery between 1997 and 2015. Major complications include wound dehiscence, postoperative infection, and ulcer recurrence.^[13]^

**Table 1:** Overview of the recurrence rate in literature for flap surgery.

Authors, published year	Patient no.	No. of sores	Recurrence rate (%)	Average period of follow-up (yr)	Average time to recurrence (d)
Keys et al 2010^[7]^	135	227	39	4.4	365 (48% in 1st year)
Larson et al 2012^[11]^	101	179	16.8	1.7	435.9
Grassetti et al 2014^[12]^	143	143	22.4	2	NA
Bamba et al 2017^[13]^	276	347	28.6	NA	357.9
Wurzer et al 2018^[14]^	55	63	27	NA	728
Firriolo et al 2018^[15]^	24	30	42	4.9 (median)	NA
Morel et al 2019^[16]^	85	85	30.6	NA	277 (median)

NA = not available.

Therefore, preoperative assessment should be comprehensive. Nutritional supplements such as sufficient albumin (>3 g/dL) are associated with less ulcer recurrence, as described by Sirimaharaj et al.^[17]^ Exudate management also maintains the quality of skin.^[18]^ Besides, postoperative wound care, such as pressure relief, nutritional supplements, and multidisciplinary approaches also matter. Jósvay et al took advantage of the fluidization bed and prone position to protect surgical sites from pressure loading to reduce the recurrence rate.^[19,20]^

The dermal components of the harvested skin graft prevented wound contraction. In 1990, Brown et al^[21]^ first described “deepithelialized full-thickness” grafts and pronounced that the amount of intact dermal collagen matrix was the key to the inhibition of contraction. The dermal matrix plays a role in wound healing by providing a scaffold for granulation tissue, stimulating angiogenesis, containing growth factors, and regulating fibroblast behavior.^[22]^ Han et al reported that dermal graft is another type of skin graft that is different from a split-thickness skin graft or full-thickness skin graft owing to superiority in its scar quality on both the recipient and donor sites when it comes to wound coverage.^[23]^ Besides, in acute burn injury, it plays a role.^[24]^ In our previous experience, with the same thickness harvested by the Zimmer dermatome, the dermal layer in the dermal graft was thicker than that in the skin graft. Dermal grafts have a pure dermis, whereas skin grafts also contain epidermal components. Moreover, the technique used to obtain dermal grafts results in lower morbidity at the donor site than skin grafts.

In this case, the decision to use a dermal graft was made because of the patient’s tendency to recurrent pressure injuries and multiple medical histories. In our experience, postoperative wound care is less challenging with a dermal graft than with flap surgery, as the recipient and donor sites are smaller and there is less tension on the wound. The use of dermal grafts has several benefits over other reconstructive methods. In addition to allowing for a second skin graft reconstruction and providing a favorable condition for subsequent grafting procedures, the dermal graft also has a smaller wound size and less tension force on the wound, making postoperative wound care less challenging. These benefits are particularly important for bedridden patients, who are at a higher risk of wound dehiscence and require a shorter healing process.

Despite the favorable preoperative parameters of our patient, including an albumin level of 3.93 g/dL and wound size of 9 cm^2^, we observed graft failure with slough formation after 1 week. This could potentially be due to poor wound conditions such as secondary infection and long-term ulceration. However, we noticed that the wound bed improved with granulation, and the wound area shrank by half of its original size after 2 months. Eventually, the wound healed after 3.5 months. The sloughing graft functioned as a wound dressing and provided beneficial effects, as described by Lee et al.^[22]^

We anticipated a simple procedure using dermal grafts would be beneficial for treating these complex wounds. However, it is important to note that this study had some limitations. First, we do not suggest that the dermal graft technique can completely replace flap surgery for the treatment of all pressure ulcers. In our case, the reconstruction with dermal grafts was applied to pressure ulcers in sacral region. Nevertheless, ischial regions, trochanters, and heels are also areas susceptible to pressure ulcers. Second, to provide further evidence for the effectiveness of dermal grafts, larger case numbers are required in future studies. Moreover, long-term follow-up monitoring of wound healing outcomes is essential.

## 5. Conclusion

In this particular case, we employed dermal grafts as an innovative method for reconstructing pressure ulcer wound sites, which has not been previously reported. Our observations indicated that the use of dermal grafts led to improved wound bed conditions and eventually resulted in wound healing, even in instances where the graft may have failed.

## Author contributions

**Conceptualization:** Yun-Nan Lin.

**Data curation:** Te-Wei Cheng.

**Investigation:** Yun-Nan Lin.

**Methodology:** Yun-Nan Lin.

**Project administration:** Yun-Nan Lin.

**Supervision:** Su-Shin Lee, Yur-Ren Kuo.

**Writing – original draft:** Te-Wei Cheng.

**Writing – review & editing:** Te-Wei Cheng, Yun-Nan Lin.
